# The Year Book of Treatment for 1890
*The Year Book of Treatment for 1890. (Messrs. Cassell and Co. Limited.)


**Published:** 1890-03-08

**Authors:** 


### PRACTITIONERS' BOOKSHELF.

### THE YEAR BOOK OF TREATMENT FOR 1890.*

"The object of this book is to present to the Practitioner not only a complete account of all the more important advances made in the treatment of disease, but to furnish also a Review of the same by competent authorities," and we find on perusing the issue for this year of this most useful and valuable compendium, that this statement in the preface is fully justified. The list of contributors on the title-page is sufficient evidence of the authoritativeness of the views to be met with there, containing as it does the names of Dr. Mitchell Bruce, who deals with diseases of the heart and circulation; Dr. Markham Skerritt who gives the new remedies for lung diseases, Drs. Ross and Reynolds, Sir Dyce Duckworth, Dr. Robert Maguire, Dr. Ralfe, Dr. Goodhart, Dr. Sydney Phillips, Mr. Frederick Treves, Dr. Walsham, Mr. Edward Owen, Mr. Reginald Harrison, Mr. Alfred Cooper, Dr. Berry Hart, Dr. Herman, Mr. Malcolm Morris, Mr. Henry Power, Mr. Field, and Dr. McBride, who all write on their respective specialities. An excellent summary of the therapeutics for the year by Dr. Walter G. Smith, Dublin, brings the work to a conclusion. We have recently tested the book in a variety of cases and found it just what was wanted. If gives the most recent treatment of disease, and at the same time the restraining hand of the experienced clinical teacher is to be felt, when that treatment is not yet firmly established, and is still under review. We wish that such a *multum in parvo* had been available when we first were launched in practice. It would have saved much wading through Reviews, Proceedings, and such like, for it not only gives concisely the ideas of the writers of the papers, but also the references to these papers, and, as we note above, a commentary by a practised hand. We may add that the book is a comfortable one to use, has a good index, and the type judiciously arranged, so that the most important points are brought quickly under the eye. We welcome this invaluable publication which ought to be found in every doctor's library.

* The Year Book of Treatment for 1890. (Messrs. Cassell and Co. Limited.)

